# Supplementary material for: Empirical methods for controlling false positives and estimating confidence in ChIP-Seq peaks
Source: BMC Bioinformatics. 2008 Dec 5;9:523. doi: 10.1186/1471-2105-9-523 (PMC2628906; doi:10.1186/1471-2105-9-523)
Supplement: Additional File 1 — A variety of html documents from the USeq web site detailing the available applications, their best usage, output file type descriptions, command line menus, etc. [file 1471-2105-9-523-S1.zip › USeqUserGuides/JavaDoc.zip!/JavaDoc/trans/roc/class-use/Positive.html]

Uses of Class trans.roc.Positive


|  |  |  |
| --- | --- | --- |
| **Overview** \| **Package** \| **Class** \| **Use** \| **Tree** \| **Deprecated** \| **Index** \| **Help** | |  |
| PREV   NEXT | **FRAMES**    **NO FRAMES**     **All Classes** |  |


---


## **Uses of Class trans.roc.Positive**

| Packages that use Positive | |
| --- | --- |
| **trans.graphics** | For graphically representing '.cel' files and intervals. |
| **trans.roc** | For manipulating the BAC dmel spike in data and generating ROC curves. |

Uses of Positive in trans.graphics

| Methods in trans.graphics with parameters of type Positive | |
| --- | --- |
| `static void` | `AggregatePlots.loadRegions(Sgr sgr, Positive[] pos)`             Checks to see if an sgr object is in the positives, if so, adds the sgr object to the positive. |
| `static int` | `AggregatePlots.maxLength(Positive[] regions)`             Finds max length of the Region[], stop-start+1 |

Uses of Positive in trans.roc

| Methods in trans.roc that return Positive | |
| --- | --- |
| `static Positive[]` | `Positive.filter(int bpOffSetEnds, Positive[] pos)`             Adds the bpOffSetEnds to the end of each Positive. |
| `static Positive[]` | `ParseSgrsForParticularRegions.parseRegionFile(java.io.File picksFile)` |

| Methods in trans.roc with parameters of type Positive | |
| --- | --- |
| `static Positive[]` | `Positive.filter(int bpOffSetEnds, Positive[] pos)`             Adds the bpOffSetEnds to the end of each Positive. |
| `static int` | `ScoreParsedBars.inRegion(Sgr sgr, Positive[] pos)`             Checks to see if an sgr object is in one of the positives, if not returns -1, otherwise returns index number. |
| `static int` | `ParseSgrsForParticularRegions.inRegion(Sgr sgr, Positive[] pos)`             Checks to see if an sgr object is in one of the positives, if not returns -1, otherwise returns index number. |
| `void` | `ScoreParsedBars.loadRegion(Positive region)`             Loads a Positive with Gr objects. |
| `static void` | `ParseSgrsForParticularRegions.loadRegions(Sgr sgr, Positive[] pos)`             Checks to see if an sgr object is in the positives, if so, adds the sgr object to the positive. |
| `void` | `ScoreParsedBars.makeRandom(Positive region, double numberScores)`             Takes a Positive and finds 1000 chrom, length, gc, and number scores matched random regions. |
| `static int` | `SgrBacCounter.positive(Positive[] pos, Sgr sgr)`             Checks to see if an sgr object is one of the positives, if not returns -1, otherwise returns index number. |
| `static int` | `MethodTester.positive(Positive[] pos, Sgr sgr)`             Checks to see if an sgr object is one of the positives, if not returns -1, otherwise returns index number. |

---


|  |  |  |
| --- | --- | --- |
| **Overview** \| **Package** \| **Class** \| **Use** \| **Tree** \| **Deprecated** \| **Index** \| **Help** | |  |
| PREV   NEXT | **FRAMES**    **NO FRAMES**     **All Classes** |  |


---
